# Engineering the anthocyanin regulatory complex of strawberry (*Fragaria vesca*)

**DOI:** 10.3389/fpls.2014.00651

**Published:** 2014-11-19

**Authors:** Kui Lin-Wang, Tony K. McGhie, Mindy Wang, Yuhui Liu, Benjamin Warren, Roy Storey, Richard V. Espley, Andrew C. Allan

**Affiliations:** ^1^The New Zealand Institute for Plant and Food Research LimitedAuckland, New Zealand; ^2^Plant and Food Research LimitedPalmerston North, New Zealand; ^3^Gansu Key Lab of Crop Improvement and Germplasm Enhancement, Gansu Agricultural UniversityLanzhou, China; ^4^Plant and Food Research LimitedTe Puke, New Zealand; ^5^School of Biological Sciences, University of AucklandAuckland, New Zealand

**Keywords:** *Fragaria vesca*, *FvMYB10*, transcription factor, regulation, anthocyanin, strawberry

## Abstract

The woodland strawberry, *Fragaria vesca* is a model fruit for a number of rosaceous crops. We have engineered altered concentrations of anthocyanin in *F. vesca*, to determine the impact on plant growth and fruit quality. Anthocyanin concentrations were significantly increased by over-expression or decreased by knock-down of the R2R3 *MYB* activator, *MYB10*. In contrast, a potential *bHLH* partner for *MYB10* (*bHLH33*) did not affect the anthocyanin pathway when knocked down using RNAi constructs. Metabolic analysis of fruits revealed that, of all the polyphenolics surveyed, only cyanidin, and pelargonidin glucoside, and coumaryl hexose were significantly affected by over-expression and knock down of *MYB10*. Using the *F. vesca* genome sequence, members of the *MYB*, *bHLH*, and *WD40* families were examined. Global analysis of gene expression and targeted qPCR analysis of biosynthetic genes and regulators confirmed the effects of altering *MYB10* expression, as well as the knock-down of *bHLH33*. Other members of the MYB transcription factor family were affected by the transgenes. Transient expression of strawberry genes in *Nicotiana benthamiana* revealed that MYB10 can auto-regulate itself, and potential repressors of MYB10. In tobacco, MYB10's activation of biosynthetic steps is inhibited by the strawberry repressor MYB1.

## Introduction

The woodland strawberry, *Fragaria vesca* (2n = 2x = 14), has a small genome (240 Mb) (Folta and Davis, 2006), and a short generation time. It is easy to propagate both from seed and clonally, and can be efficiently transformed by *Agrobacterium*-mediated transformation (Oosumi et al., 2006) to enhance secondary plant products (Bulley et al., 2012). Therefore, it has become a model plant for the diverse *Rosaceae* family, which contains many valuable fruit and ornamental crops (Folta and Dhingra, 2006).

Strawberry fruits are distinguished by anthocyanins, the water-soluble flavonoid pigments that are found in higher plants and are responsible for the red, purple, and blue color of many fruits, flowers, leaves and seeds. There is growing evidence from epidemiological, *in vitro* and *in vivo* studies for the role of anthocyanins in potential long-term health benefits and an association with reduced incidence of chronic diseases, although there are still many questions regarding the mechanistic action and specific health effects (Traka and Mithen, 2011). Anthocyanins have been the target of a number of recent studies concerning the improvement of the dietary health benefits of fruit (Davies and Espley, 2013). Intervention trials using highly anthocyanic maize, tomato, and apple have all shown a number of positive health effects in small mammals (Butelli et al., 2008; Toufektsian et al., 2008; Espley et al., 2014). The evidence makes a strong case for further investigation and the development of foods with high concentrations of bioactive secondary metabolites, regardless of the breeding approach employed. One method might be the manipulation of secondary metabolite pathway regulation.

In all species studied to date, the regulation of anthocyanin biosynthesis is at the level of transcriptional regulation of genes encoding enzymes of the biosynthetic steps by transcription factors that include MYB, bHLH, and WD-repeat proteins (the MYB-bHLH-WD40 “MBW” complex, Baudry et al., 2006; Allan et al., 2008; Butelli et al., 2008; Jaakola, 2013). MYB genes are identified by their conserved DNA-binding domain consisting of one or more repeats and those that control anthocyanin are highly conserved amongst many species examined and fall into a specific subgroup (Allan et al., 2008). The MYB genes involved in the “MBW” complex are the two repeat R2R3 class. There are both activating and repressing R2R3 MYBs. Over-expression of the MYB member of the complex has dramatic effects. In *Arabidopsis*, over-expression of *AtPAP1* or *AtPAP2* results in the accumulation of anthocyanin (Gonzalez et al., 2008). The concentration of anthocyanin is substantially elevated by over-expressing both bHLH (*Del*) and MYB (*Ros1*) genes from the snapdragon *Antirrhinum majus* in the fruit of transgenic tomatoes (Butelli et al., 2008). In one of these transgenic tomato lines the highest concentration of 2.83 ± 0.46 mg of anthocyanin per g fresh weight was measured, while anthocyanins were virtually undetectable in wild-type tomato fruit. In apple, overexpression of *MdMYB10* in “Royal Gala,” a white-fleshed, green-leaved apple cultivar results in a red leaf and fruit phenotype (Espley et al., 2007). Furthermore, environmental factors (i.e., temperature and light) can alter the red coloration of *Arabidopsis* over-expressing *AtPAP1* plants (Rowan et al., 2009). High temperatures (30–37°C) have been shown to decrease anthocyanin content in the skin of apple (Lin-Wang et al., 2011).

Repressors of anthocyanin accumulation were also identified within the MYB family of transcription factors, including *AtMYB3*, *AtMYB4*, and *AtMYBL2* from *Arabidopsis* (Jin et al., 2000; Dubos et al., 2008), *MdMYB16*, *MdMYB17*, and *MdMYB111* from apple (Lin-Wang et al., 2011), and *FaMYB1* and *FcMYB1* from strawberry (Aharoni et al., 2001; Salvatierra et al., 2013). It was reported that flowers of transgenic tobacco lines overexpressing *FaMYB1* showed a severe reduction in pigmentation, and a reduction in the concentration of cyanidin 3-rutinoside (an anthocyanin) and quercetin-glycosides (flavonols) was detected (Aharoni et al., 2001). Another MYB family member *FaMYB5* was also described as a putative negative regulator in the strawberry proanthocyanidin biosynthesis pathway (Schaart et al., 2013).

In the *Fragaria* genus, fruit color is determined by the accumulation of anthocyanin, the most abundant flavonoid-derived component in strawberry fruits (Hannum, 2004). Over-expression of the anthocyanin biosynthesis activator *FaMYB10* in *F. ananassa* results in plants with elevated root, foliar, and fruit anthocyanin concentrations (Lin-Wang et al., 2010). A recent study showed that over-expression of *Arabidopsis* proanthocyanidin (PA) biosynthesis regulators *AtTT2*, *AtTT8*, and *AtTTG1* in *F. ananassa* resulted in a partial loss of red coloration in the flesh, associated with an increased PA content and decreased anthocyanin accumulation (Schaart et al., 2013). In contrast, the silencing of the PA biosynthesis enzyme anthocyanidin reductase in strawberry resulted in a redirection of flavonoid production away from PAs and an increase in anthocyanin production during early fruit development (Fischer et al., 2014) whilst silencing *FaGT1*, the 3-0-glucosyltransferase implicated in glycosylating anthocyanidins, has been shown to specifically reduce anthocyanins with a concomitant increase in flavan-3-ols (Griesser et al., 2008).

In this study we used the diploid strawberry *F. Vesca* as a model for the related octoploid cultivated strawberry, *F. ananassa*. *F. Vesca* benefits from a relatively small genome, good transformation efficiency, and ease of propagation. We describe how over-expression of *FvMYB10* in *F. vesca* produced purple-skinned and red-fleshed strawberries, while knock down of *FvMYB10*, using an RNAi construct produced white-skinned and white-fleshed fruit. The phenotypic, metabolomic and expression data for the stable transgenic lines 35S:*FvMYB10* and *FvMYB10* RNAi confirmed that *FvMYB10* plays a key role in activating anthocyanin biosynthesis pathway in *F. vesca*. Stable transformation in this model rosaceous species provides information on plant performance in the presence of altered levels of anthocyanins and suggests the possibility of developing novel strawberry crops by traditional or non-traditional breeding technologies. This information can further be translated to other rosaceous fruit crops to help to understand the effect manipulating metabolite levels.

## Materials and methods

### Strawberry transformation

“Alpine” strawberry *F. vesca* ssp. *vesca* was selected for gene isolation and plant transformation. For overexpression of *FvMYB10*, the full-length cDNA of *FvMYB10* was cloned into the pSAK277 vector (Gleave, 1992). For knock down expression of *FvMYB10* and *FvbHLH33*, cDNA fragments corresponding to nucleotides 171–636 and 609–1088 of *FvMYB10* and *FvbHLH33*, respectively (relative to the ATG start codon) were selected. Sequences were blasted to the strawberry genome (http://www.rosaceae.org/tools/ncbi_blast) to confirm the specificity to the gene of interest only. The *FvMYB10* fragment was cloned using the 5′ to 3′ primer sequences GCCGAATATCAAGAGAGGAGAGTTT (forward) and GAAGTCATGATCCTCCAAACCAATA (reverse). The *FvbHLH* fragment was cloned using the 5′ to 3′ primer sequences AAAGCCAGATTGCTCCGAGAAA (forward) and AGACCCAAGGAGCTGAACTTTGTAT (reverse). The fragments were cloned into a commercial vector pENTR/D–TOPO (pENTR Directional TOPO Cloning Kits, Life Technologies) respectively. Two copies of the selected gene fragment were inserted into an RNAi vector pTKO2 in an inverse orientation, flanking an intron sequence, using a Gateway LR reaction (Snowden et al., 2005). The construct was electroporated into *Agrobacterium tumefaciens* GV3101, then transformed into *F. vesca*, using the *Agrobacterium*-mediated strawberry transformation protocol as previously described (Oosumi et al., 2006).

### Liquid chromatography-quadrupole-time of flight-mass spectrometry analysis of anthocyanins

Samples of frozen ground fruit, approximately 150 mg, were extracted overnight at 1°C with 1.0 mL solvent (ethanol/water/formic acid 80/20/1 v/v/v). The samples were centrifuged, diluted and analyzed by liquid chromatography and quadrupole time of flight, high resolution, mass spectrometry (LC-QTOF-HRMS). The LC-MS system was composed of a Dionex Ultimate® 3000 Rapid Separation LC and a micrOTOF QII high resolution mass spectrometer (Bruker Daltonics, Bremen, Germany) fitted with an electrospray ion source. Component separation by LC was achieved using two UHPLC columns connected in series. The columns were both Zorbax™ SB-C18 2.1 × 100 mm, 1.8 μm (Agilent, Melbourne, VIC, Australia) and were maintained at 60°C. The flow was 350 μL/min. The solvents were A = 100% acetronitrile, and B = 0.4% formic acid. The solvent gradient was: 10% A, 90% B, 0–0.5 min; linear gradient to 100% A, 0.5–25 min; composition held at 100% A, 25–28 min; linear gradient to 10% A, 90% B, 28-28.2 min; to return to the initial conditions before another sample injection at 31 min. The injection volume for samples and standards was 1 μL. The micrOTOF QII parameters for polyphenolic analysis were: temperature 225°C; drying N_2_ flow 6 L/min; nebulizer N_2_1.5 bar, endplate offset −500 V, mass range 100–1500 Da, acquired were acquired at 2 scans/s. Negative ion electrospray was used with a capillary voltage of +3500 V. Post-acquisition internal mass calibration used sodium formate clusters, with the sodium formate delivered by a syringe pump at the start of each chromatographic analysis. Polyphenols compounds were quantified using QuantAnalysis (Bruker Daltonics, Bremen, Germany) software. To quantify target compounds, exact (± 10 mDa) ion chromatograms (EICs) for each of the target compounds were extracted from the three dimensional LCMS data of each sample and calibration standard. The concentrations of components in samples were calculated by comparison with external calibration curves of authentic compounds. When an authentic compound was not available, the calibration curve of a similar compound was used to calculate equivalents. For example all anthocyanins were quantified as cyanidin 3-glucoside equivalents.

### Real-time qPCR expression analysis

Mature strawberry fruit were collected from the over-expressing lines, RNAi lines and wild-type controls. Total RNA from three biological replicates per time point was extracted, using a method adapted from Chang et al. (1993). Removal of genomic DNA contamination and first-strand cDNA synthesis were carried out using oligo (dT) according to the manufacturer's instructions (QuantiTect Reverse Transcription Kit, Qiagen). Real-time qPCR DNA amplification and analysis was carried out using the LightCycler 480 Real-Time PCR System (Roche), with LightCycler 480 software version 1.5. The LightCycler 480 SYBR Green I Master Mix (Roche) was used following the manufacturer's method. The qPCR conditions were 5 min at 95°C, followed by 45 cycles of 5 s at 95°C, 5 s at 60°C, and 10 s at 72°C, followed by 65–95°C melting curve detection. The qPCR efficiency of each gene was obtained by analyzing the standard curve of a cDNA serial dilution of that gene. Five reference genes *ACTIN* (XM_004307470), *GAPDH* (XM_004309993), *PP2a* (XM_004297079), *SAND* (gene21567-v1.0-hybrid, Genome Database for Rosacea), and *UBC9* (XM_004307970) were examined. *GAPDH* (XM_004309993), *PP2a* (XM_004297079), and *UBC9* (XM_004307970) were selected because of their consistent transcript levels throughout samples, with crossing threshold values changing by less than 2. Transcript abundance was calculated relative to the geometric mean of *GAPDH*, *PP2a*, and *UBC9* (Pfaffl, 2001). All primer sequences are listed in Supplementary Table 1.

### Phylogenetic analysis

The candidate genes *FvMYB1*, *FvMYB5*, *FvbHLH3*, *FvbHLH33*, *FvMYC1*, and *FvTTG1* were selected by blasting protein sequences of published corresponding genes in *F. ananassa* to “Non-redundant protein sequences (nr)” in NCBI protein database, and then taking the highest-scoring genes (in our case, *E* values were less than 8e-126) and then performing a reciprocal blast back to the NCBI protein database to confirm the original genes had the highest scores. Protein sequences were aligned using Blosum62 (Gap open penalty: 90 and Gap extension penalty: 3) in Geneious 6.1.7. Phylogenetic trees were generated using MEGA 6.06 and the Neighbor-Joining method with 1000 bootstrap replicates.

### Dual luciferase assay of transiently transformed *Nicotiana benthamiana* leaves

A fragment containing around 1500 bp upstream of the start code ATG of each candidate gene was isolated by PCR, and inserted into the cloning site of pGreenII 0800-LUC (Hellens et al., 2005), and modified to introduce an *NcoI* site at the 3′ end of the sequence. This allowed the promoter to be cloned as a transcriptional fusion with the firefly luciferase gene (LUC). The promoter-LUC fusion in pGreenII 0800-LUC was used in transient transformation by mixing 100 μl of *Agrobacterium* strain GV3101 (MP90) transformed with the reporter cassette with 300 μl each of three other *Agrobacterium* cultures. These three cultures had been transformed with cassettes containing a cDNA of MYB TF gene or a bHLH TF gene fused to the 35S promoter, respectively, in either pSAK277 (Gleave, 1992), or pHex2 (Hellens et al., 2005). *N. benthamiana* growing conditions, *Agrobacterium* infiltration processes and luminescent measurements were as described by Hellens et al. (2005).

### Deep sequencing and transcriptome analysis

Four RNA-seq libraries from the pooled mature fruit of 35S:*FvMYB10-2*, *FvMYB10*RNAi-2, *FvbHLH33*RNAi-1, and WT-4 were prepared from total RNA using a method adapted from Chang et al. (1993). cDNA library construction from total RNA and next generation sequencing (NGS) were carried out at Macrogen, Inc., Korea. HiSeq2000 platform was implemented and the libraries were sequenced as 100 bp paired-end reads. Around 70 million reads were generated from each library. Deep sequencing reads were quality assessed with the quality assessment software FastQC (Blankenberg et al., 2010). The adaptor sequences were removed for all the reads. The reads were trimmed (15 bp from the 5′ end, and 5 bp from the 3′ end) and quality filtered (quality cut-off value: 20, percentage of bases in sequence that must have quality equal to/higher than cut-off value: 90). The reads were mapped to the reference “fvesca_v1.0_genemark_hybrid.annotated.fna” (downloaded from Genome Database for Rosaceae, www.rosaceae.org) with BWA (Li and Durbin, 2009). Over 60% reads were successfully mapped to the reference. RPKM (reads per kilobase per million mapped reads) was estimated with Cufflinks (Trapnell et al., 2010). Hierarchical cluster analysis of *FvMYBs* expression based on normalized RPKM value was plotted using a custom R script.

### Headspace volatile analysis by GC-MS

Mature strawberry fruit were harvested from green house at 10 to 11 am during November to December 2013. For each of three replicates, about 20 grams of the fruit (more than 20 strawberries) was weighed in a 250 mL conical flask. Headspace volatiles from the flask were collected with the purge and trap method according to Wang et al. (2011). Volatiles above the fruit were blown with 25 mL min^−1^ of purified air flow and concentrated onto an absorbent column packed with 80 mg Tenax^*TA*^ 60/80 (Alltech) attached to an outlet of the system. Sample collection time was 3 h at room temperature. Ethyl ester (0.2 mL) was added to the top of the absorbent column three times. Eluate (0.5 mL) was collected from the column at a low pressure with a pre-weighed 1.5 mL auto-GC injection vial. The ether extract elute was weighed and 2μL of 0.9 μg μL^−1^ of hexadecane was added as the internal standard before GC-MS analysis. Ether elute was analyzed with GC-MS (7890A–5975C, Agilent Technologies) with a 50:1 split ratio. One μL of the ether elute was injected into the GC at 250°C (Column: Restek Rxi-5 ms, 30 m × 250 μm × 0.25 μm). The oven temperature was 60°C for 2 min; then 6°C min^−1^ to 120°C; 10°C min^−1^ to 200°C; 30°C min^−1^ to 250°C; 60°C min^−1^ to 300°C; then hold 300°C for 3 min. MS acquisition scan mode was set with low mass = 35 and high mass = 400. EM voltage was 1212 volts. MS source temperature was 230°C. Peaks from the sample were integrated with MSD ChemStation E02.02.1431. The mass spectrometry trail of each peak was identified by comparing with the standard spectra recorded in the Nist05a library or the in-house authentic standards and assisted with compound Kovats retention index. The amount of each compound in the fruit (μg g^−1^ of the fresh fruit) was calculated with the standard curves prepared with a standard solution with known deferent concentration of ethyl butanoate, ethyl hexanoate, alpha pinene, and beta-myrcene prepared for strawberry fruit.

### Statistics

For qPCR analysis, data are presented as means (± *SE*) of three biological replicates. For transient transformation promoter activation assays, data are presented as means (± *SE*) of four biological replicates. For the analysis of anthocyanins, polyphenols and volatile compounds, data are presented as means (± *SE*) of three biological replicates. Statistical significance was determined by one-way ANOVA.

## Results

### Generation of 35S:*FvMYB10, FvMYB10* RNAi, and *FvbHLH33* RNAi transgenic strawberry lines

**Figure 1:**
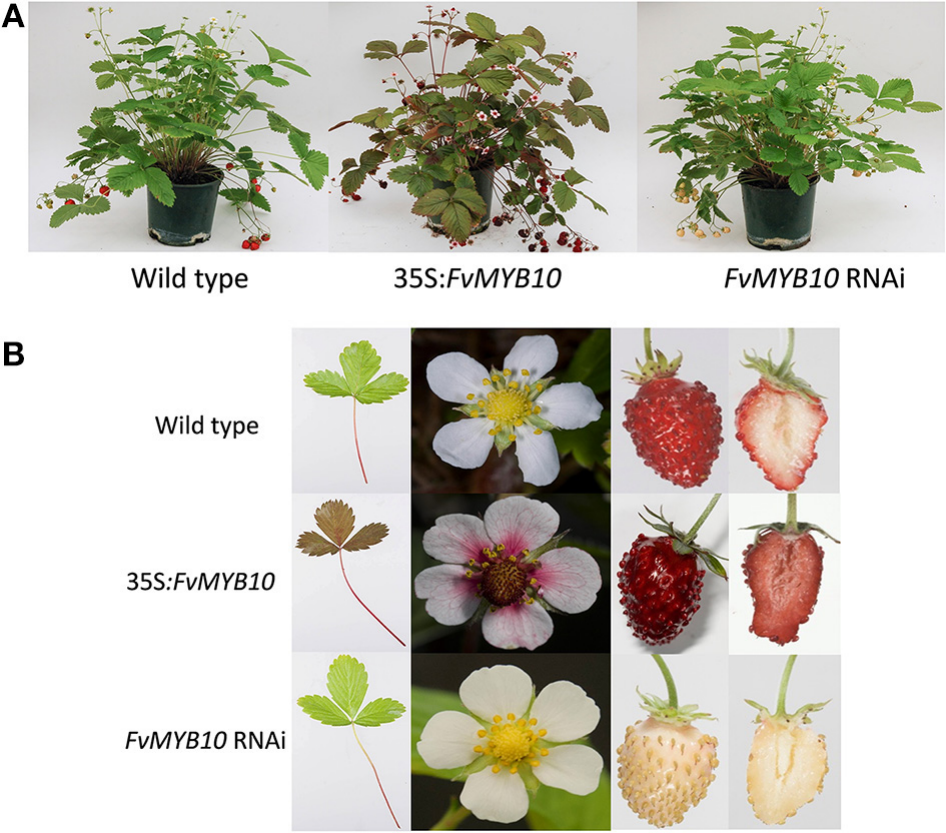
**Phenotype of strawberry plants transformed with 35S:*FvMYB10*, *FvMYB10* RNAi, and wild-type controls**. Growth and pigmentation of strawberry plants transformed with 35S:*FvMYB10*, *FvMYB10* RNAi, and wild-type controls **(A)**. Detailed phenotype of strawberry leaves, flowers and fruit of 35S:*FvMYB10*, *FvMYB10* RNAi, and wild-type controls. Lines of 35S:*FvMYB10* had pigmented leaves, petioles, stigmas and petals, and mature fruit had dark red/purple skin and red flesh. The mature fruit of *FvMYB10* RNAi lines had white skin and white flesh, and the only pigmented tissue was the petioles **(B)**.

**Figure 2:**
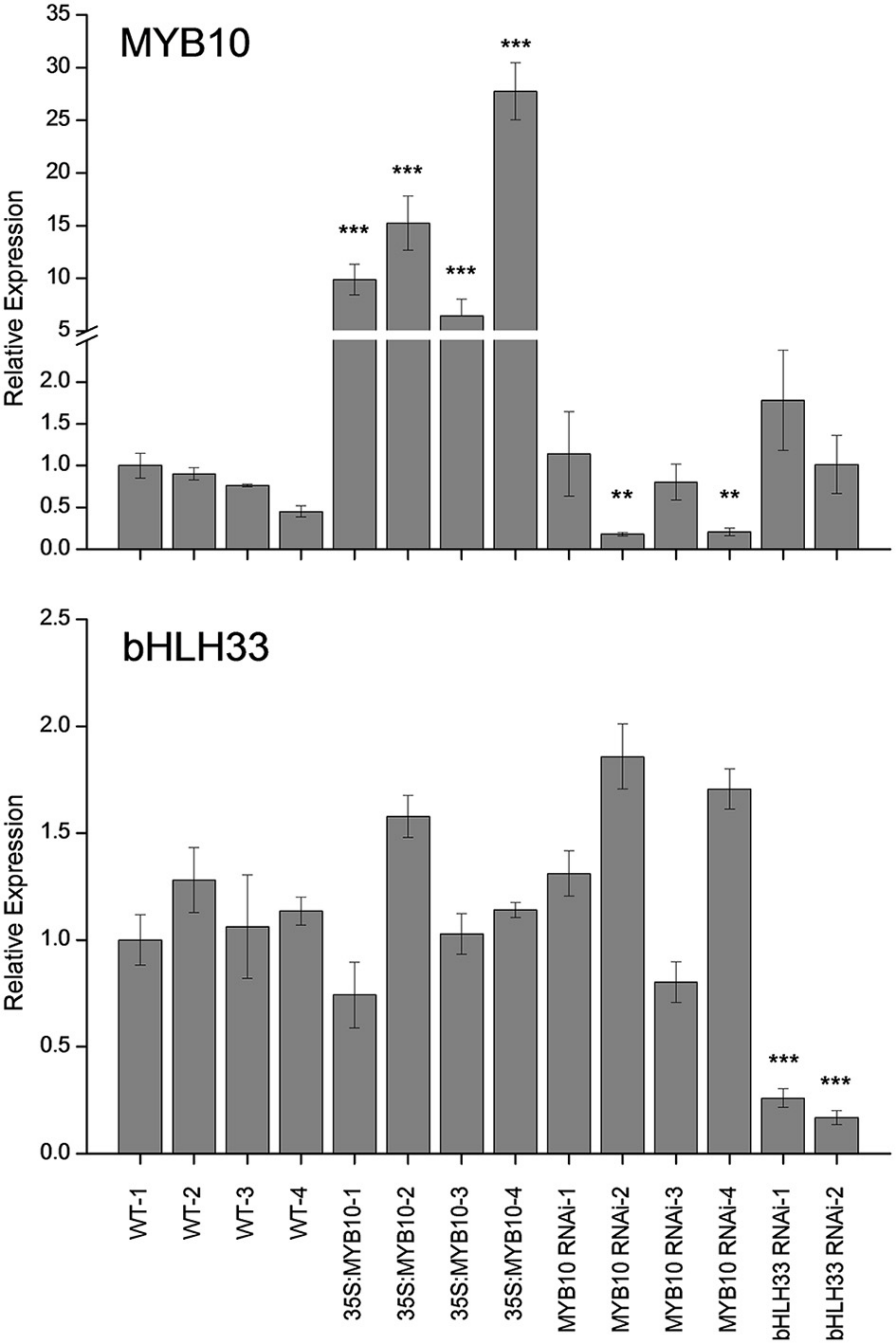
**Expression analysis of *FvMYB10* and *FvbHLH33* in 35S:*FvMYB10*, *FvMYB10* RNAi, *FvbHLH33* RNAi, and wild type-controls**. The expression of *FvMYB10* increased by 6 to 30-fold in all 35S:*FvMYB10* lines, and decreased by 3 to 6-fold in two *FvMYB10* RNAi lines (*FvMYB10* RNAi-2 and *FvMYB10* RNAi-4). The transcript of *FvbHLH33* was significantly reduced in two *FvbHLH33* RNAi lines. Transcript abundance was calculated relative to the geometric mean of *GAPDH*, *PP2a*, and *UBC9*. Errors bars are the *SE* for three biological replicates. Statistical significance between transgenic lines and wild-type controls was determined by One-Way ANOVA: ^*^P < 0.05; ^**^P < 0.01; ^***^P < 0.001.

*F. vesca* was transformed with a construct containing 35S:*FvMYB10*. Leaves, petioles, stigmas, and petals of the 35S:*FvMYB10* lines were pigmented, and mature fruit of these lines had dark red/purple skin and red flesh, compared with red skin and white flesh in wild-type mature fruit (Figure 1). All the 35S:*FvMYB10* lines showed the red phenotype, although the degree of red coloration varied among the four transgenic lines. A previous study showed that when 35S:*FaMYB10* was transformed into *F. ananassa*, plants were not highly pigmented, although mature fruit from 35S:*FaMYB10* lines showed approximately 50% more anthocyanin than wild-type controls (Lin-Wang et al., 2010). The transcript level of *FvMYB10* expression in the four 35S:*FvMYB10* lines were elevated by 6 to 30-fold compared with that in wild-type controls (Figure 2).

The mature fruit of *FvMYB10* RNAi lines had white skin and white flesh and the only pigmented tissue in these RNAi lines was the petiole (Figure 1). Four *FvMYB10* RNAi lines were generated in this study, with only two lines (*MYB10* RNAi-2 and *MYB10* RNAi-4) having white fruit and two lines (*MYB10* RNAi-1 and *MYB10* RNAi-3) showed pigmented fruit comparable to wild type (WT) (not shown in figures). In contrast, the mature fruit of two lines of *FvbHLH33* RNAi lines showed unaltered pigmentation compared to WT (not shown in figures). To determine the efficiency of the RNAi knock-down constructs, quantitative real-time PCR (qPCR) was performed on all lines (Figure 2). This shows that in the white-fruited lines (*MYB10* RNAi-2 and *MYB10* RNAi-4) there was the lowest expression of *MYB10*. This also correlated with a slight increase in *bHLH33* transcript. Although the *FvbHLH33* RNAi lines showed unaltered pigmentation phenotype compared to WT, the transcript of *bHLH33* was significantly reduced.

### Liquid chromatography-quadrupole-time of flight-mass spectrometry analysis of anthocyanins and polyphenols

**Figure 3:**
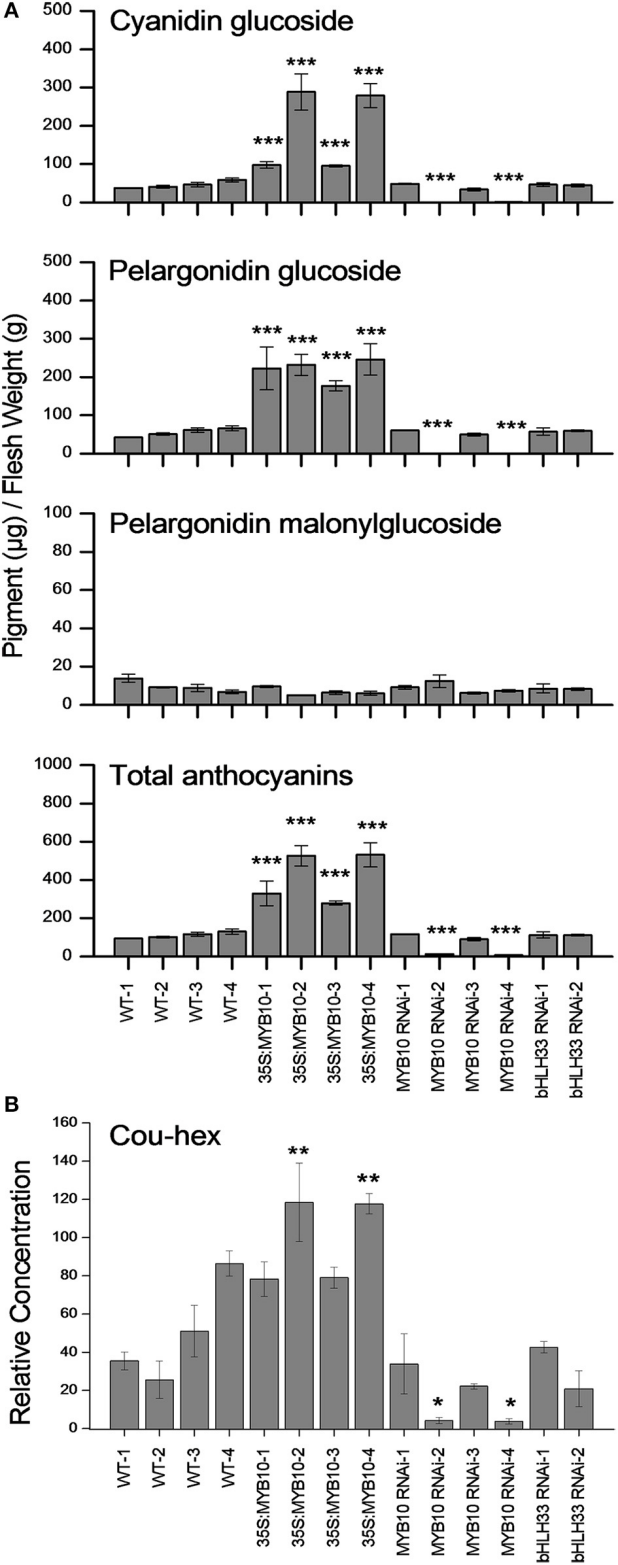
**Concentrations of anthocyanins and p-coumaryl glucose in the mature fruit of 35S:*FvMYB10*, *FvMYB10* RNAi, *FvbHLH33* RNAi, and wild-type controls**. The two main anthocyanins, cyanidin glucoside and pelargonidin glucoside, were elevated in 35S:*FvMYB10* lines and barely detectable in two *FvMYB10* RNAi lines. The third anthocyanin compound, pelargonidin malonylglucoside, remained at low concentrations in all fruit. There was no significant difference in anthocyanin concentration between *FvbHLH33* RNAi lines and wild-type controls **(A)**. P-coumaryl glucose (Cou-hex) correlated with two main anthocyanin compounds (cyanidin glucoside and pelargonidin glusoside) **(B)**. Errors bars are the *SE* for three biological replicates. Statistical significance between transgenic lines and wild-type controls was determined by One-Way ANOVA: ^*^P < 0.05; ^**^P < 0.01; ^***^P < 0.001.

Pigmentation of the mature fruit in most strawberry cultivars (*F. ananassa*) is mainly due to the accumulation of pelargonidin glucoside and a small fraction of cyanidin glucoside (Fait et al., 2008). In this study, we used LC-MS to show that anthocyanin in the mature fruit of wild strawberry (*F. vesca*) is largely due to the accumulation of both cyanidin glucoside and pelargonidin glucoside and a small fraction of pelargonidin malonylglucoside (Figure 3A). There were large increases in both cyanidin glucoside and pelargonidin glusoside (2 to 5-fold) in the 35S:*FvMYB10* over-expressing lines. These two compounds were almost undetectable in two *FvMYB10* RNAi knock-down lines (*FvMYB10* RNAi-2 and *FvMYB10* RNAi-4) which at maturity had white fruit. The concentrations stayed at a similar level to that in wild-type controls in the other two *FvMYB10* RNAi knock-down lines (*FvMYB10* RNAi-1 and *FvMYB10* RNAi-3) which showed unaltered pigmentation when compared to WT (Figure 3A). The accumulation of anthocyanin was not affected by *FvbHLH33* RNAi knock-down. There were no significant changes in pelargonidin malonyl glucoside across all the transgenic lines and wild-type controls. However, the amount of this compound detected in the mature fruit was much lower than amounts of the other two compounds.

The LC-MS data for other polyphenols are shown in Supplementary Figure 1 and Supplementary Table 2. Of all the compounds detected, only p-coumaryl glucose (Cou-hex) had a similar concentration profile to that of the two main anthocyanin compounds (cyanidin glucoside and pelargonidin glusoside) (Figure 3B). A similar effect on p-coumaryl glucose was seen in *F. ananassa* fruit when FaMYB10 was transiently knocked-down (Medina-Puche et al., 2014).

### Phylogenetic analysis of homologous transcriptional regulators in anthocyanin biosynthetic pathway

**Figure 4:**
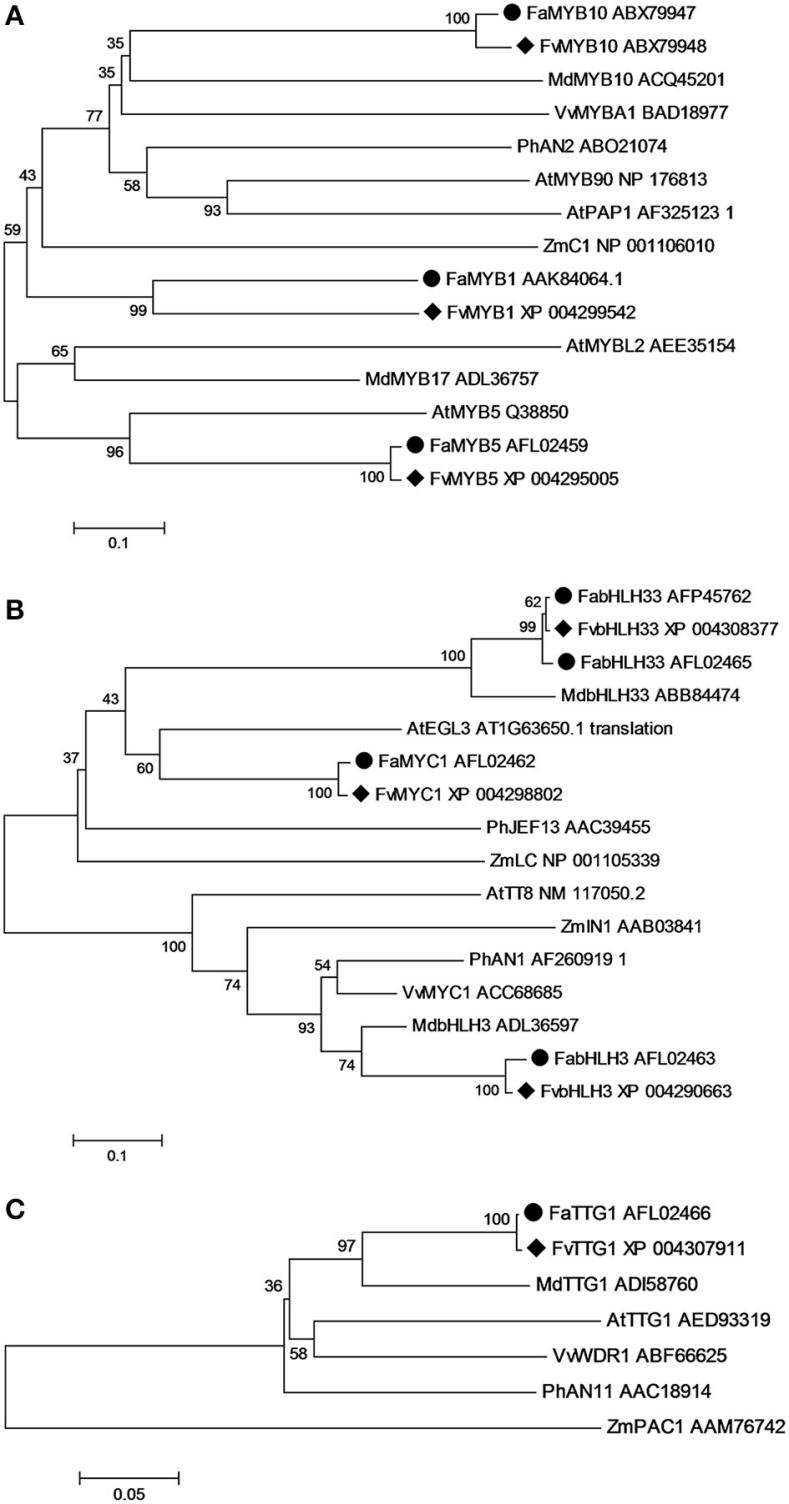
**Phylogenetic relationships of homologous putative transcriptional regulators involved in anthocyanin biosynthetic pathway from *Arabidopsis*, *Malus* x *domestica*, *Petunia* x *hybrida*, *Zea mays*, *Vitis vinifera*, and *Fragaria***. Protein sequences of MYB **(A)**, basic helix-loop-helix (bHLH) **(B)** and TTG1-like WD40 **(C)** were analyzed using MEGA 6.06 and the Neighbor-Joining method with 1000 bootstrap replicates. ♦ indicates genes from *F. vesca* and • indicates genes from *F. ananassa*.

To examine potential redundancy in the genes targeted for transformation of *F. vesca*, phylogenetic relationships were determined [using MEGA 5.2 and the neighbor-joining method and with 1000 bootstrap replicates (Tamura et al., 2011)] between *Fragaria* anthocyanin-related MYB, bHLH and WD40 repeat regulatory proteins and the known homologous transcription factor (TF) genes from *Arabidopsis*, *Malus* x *domestica*, *Petunia* x *hybrida*, *Zea mays*, and *Vitis vinifera* (Figure 4). All the MYBs associated with promoting anthocyanin biosynthesis clustered within the same subgroup as *Arabidopsis* PAP1, while MYB repressors clustered outside this subgroup (Figure 4A). All the bHLH genes were divided into two subgroups: one represented by *Arabidopsis* EGL3-like bHLHs and another by *Arabidopsis* TT8-like bHLHs (Figure 4B). There was only one AtTTG1-like WD repeat gene selected from each selected species, and the WD repeat genes from dicots clustered together into one subgroup, while the monocot ZmPAC1 was located outside this subgroup (Figure 4C).

### Analysis of global gene expression reveals the genes most affected by manipulation of MYB10 levels

To ascertain the differentially expressed genes in transformed *F. vesca* fruits, four RNA-seq libraries from mature fruit of 35S:*FvMYB10*-2, *FvMYB10*RNAi-2, *FvbHLH33*RNAi-1, and wild type were prepared and sequenced. Over 290 million reads were generated. Analysis of differential expression in these libraries revealed that the transcript levels of 480 genes in 35S:*FvMYB10* lines were greater than two-fold different than those in the wild type, while the transcript levels of 1126 genes were reduced by greater than two fold (Supplementary Tables 3, 4). In the *FvMYB10*RNAi lines 1102 genes showed a higher level of transcript abundance greater than two fold, while 2243 genes were reduced by greater than two fold (Supplementary Tables 5, 6). In all these analyses, a cut-off value of greater than or equal to 10 reads per kilobase per million reads (RPKM) was applied.

A relatively simple comparison was made of these long RNA-seq lists; an analysis of the intersection between the genes that were more than two-fold up-regulated in 35S:*FvMYB10* (in comparison with wild-type fruit), and those that were also more than two-fold down-regulated in *FvMYB10* RNAi-2 (in comparison with wild-type fruit) (Table 1). Of particular note was the gene most altered in its expression level, a GST with sequence homology to *TT19* of *Arabidopsis* (54% identical at the amino acid level to TT19, AT5G17220; although 56% identical to AT3G03190, AtGST6). TT19 functions as a carrier-transporter for cyanidin-3-O-glycoside in *Arabidopsis* (Sun et al., 2012). Also affected, but less so, was a strawberry gene with greatest homology to a proton antiporter *TT12*. These findings suggest two important candidates for anthocyanin transport in strawberry fruit.

**Table 1:** **Differentially expressed genes whose expression profile is MYB10 RNAi/WT >2 and WT/35S:MYB10>2**.

**Fv gene ID**	**Description**	**Best Arabidopsis match**	**WT**	**35S MYB10**	**MYB10 RNAi**
**RPKM of Genes with the expression pattern (35S:MYB10/WT > 2 and WT/MYB10 RNAi > 2)**					
gene31672	Glutathione S-transferase, AtGSTF6,	AT3G03190 3e-68	554	4226	10
gene21346	Chalcone isomerase like	AT5G05270 5e-74	479	2946	30
gene26825	Chalcone synthase, TT4	AT5G13930 e0	281	2829	6
gene14611	Flavanone 3-hydroxylase (F3H) TT6	AT3G51240 1e-173	365	2742	24
gene15176	Dihydroflavonol 4-reductase (DFR) TT3	AT5G42800 5e-94	148	1792	4
gene26826	Chalcone synthase TT4	AT5G13930 e0	216	1753	5
gene32347	Leucoanthocyanidin dioxygenase (LDOX) TT18	AT4G22880 1e-141	286	1222	23
gene23367	Chalcone isomerase CHI, TT5	AT3G55120 3e-84	173	765	11
gene10225	Dihydrofolate reductase	AT4G24380 6e-57	67	741	3
gene09753	Phenylalanine ammonia-lyase 1 AtPAL1	AT2G37040 e0	57	653	3
gene12591	Anthocyanidin 3-O-glucosyltransferase 2 UGT78D2	AT5G17050 1e-142	160	643	4
gene31413	AtMYB113	AT1G66370 5e-49	80	366	40
gene28093	Cinnamate-4-hydroxylase AtC4H	AT2G30490 e0	92	218	5
gene22077	Acetyl-CoA carboxylase, AtACC1	AT1G36160 e0	28	204	11
gene02203	Cinnamoyl-CoA reductase	AT2G23910 6e-65	47	156	11
gene15174	Dihydroflavonol 4-reductase (DFR) TT3	AT5G42800 1e-141	56	116	0
gene30262	Solute carrier family 35	AT3G59310 2e-57	25	69	4
gene32153	Auxin-induced protein 5NG4	AT3G28050 3e-72	27	65	5
gene12150	caffeoyl-CoA O-methyltransferase (CCoAOMT)	AT1G67980 2e-78	18	39	4
gene04357	Anthocyanidin-O-glucosyltransferase	AT2G16890 4e-23	8	36	0
gene28793	Proton antiporter TT12,	AT3G59030 1e-180	4	17	1
gene04355	Anthocyanidin-O-glucosyltransferase UGT73B1	AT4G34138 3e-28	3	15	0
gene00094	Cytokinin-O-glucosyltransferase 3 (AtZOG3)	AT3G53150 1e-159	2	11	1
gene03735	WD repeat-containing protein	AT1G25422 4e-09	0	9	0
**Genes with the expression pattern (MYB10 RNAi/WT > 2 and WT/35S:MYB10> 2)**					
gene15418	17.9 kDa class II heat shock protein	AT5G12020 1e-46	131	61	299
gene30534	Non-specific lipid-transfer protein 2 (LTP 2)	AT3G18280 1e-20	66	23	221
gene00622	Cytokinin-O-glucosyltransferase 2 AtUGT85A2	AT1G22360 7e-73	8	3	168
gene30828	E3 ubiquitin-protein ligase RNF167	AT5G45290 5e-32	61	29	151
gene30826	Glycerol-3-phosphate permease, ATG3PP5	AT2G13100 6e-09	46	4	145
gene29971	Homocysteine S-methyltransferase 3	AT3G22740 1e-138	61	27	133
gene29518	Coiled-coil domain-containing protein 109A	AT2G23790 1e-95	30	10	126
gene01202	1-aminocyclopropane-1-carboxylate oxidase	AT1G05010 1e-135	14	7	101
gene15265	17.3 kDa class II heat shock protein	AT5G12020 5e-07	24	6	58
gene08191	Auxin-induced protein AtAUX2-11	AT5G43700 1e-56	11	4	56
gene10996	17.6 kDa class II heat shock protein (AtHsp17.6)	AT5G35090 1e-06	14	5	41
gene01539	Peroxidase 35	AT3G49960 1e-119	11	3	37
gene30565	Pathogenesis-related protein PR-4B	AT3G04720 3e-57	0	0	25
gene04066	Chaperone protein dnaJ	AT1G71000 3e-38	7	1	23
gene07040	RING-H2 finger protein ATL3F	AT3G16720 3e-45	7	3	23
gene30850	Short-chain dehydrogenase/reductase	AT2G47140 2e-63	0	0	15
gene25188	Unknown protein	AT1G13360 1e-18	4	1	13
gene12691	Protein phosphatase 2C 77 (AtPP2C77)	AT5G57050 1e-112	2	1	12
gene19171	lipid transport protein	AT4G32870 8e-41	2	1	12
gene12032	RING-H2 finger protein ATL1H	AT1G35330 5e-49	5	2	10
gene17749	Protein E6	AT1G03820 3e-12	1	0	10

This list also confirmed the strawberry genes of the anthocyanin biosynthetic pathway. There appeared to be two *CHS* genes affected which have sequential gene model numbers and appear adjacent to each other in the strawberry genome (Shulaev et al., 2011), suggesting a recent gene duplication. Two *CHI* genes were differentially expressed; one shares homology to a *CHI*-like gene in *Arabidopsis* while the other is most similar to the characterized *AtCHI* (*TT5*). Two *DFR* genes were differentially regulated, while another unknown reductase (gene02203) was annotated in *Arabidopsis* as a potential cinnamoyl-CoA reductase. The list also provides identification of three potential *UFGT* genes, with gene12591 being the most likely 3-O-glucosyltransferase as it is 55% identical to At5g17050, which specifically glucosylates the 3-position of flavonoids in *Arabidopsis*.

Of the potential regulators, *MYB10* is present in the list (gene31413). However, the WD40 protein identified in this list (gene03735) has very low homology to members of the TTG1 WD40 proteins, so is unlikely to be part of the MBW complex. Four genes unrelated to anthocyanin biosynthesis are on this list, including a dihydrofolate reductase and an acetyl-CoA carboxylase (potentially involved in production of waxes and lipid metabolism).

The opposite comparison was also made; the intersection between the genes that were more than two-fold up-regulated in *FvMYB10*RNAi-2 (in comparison with wild-type fruit), and were also more than two-fold down-regulated in 35S:*FvMYB10* (in comparison with wild-type fruit). The identified genes were all unrelated to anthocyanin metabolism (Table 1). The most affected genes were several small heat shock proteins, as well as several related to stress (pathogen response, peroxidises). An ethylene-related ACO-like gene was up-regulated in white fruit. This suggests that the white fruit may be undergoing a greater stress response during ripening, possibly because of the absence of anthocyanin. Two potential transcription factors were affected: gene08191, which is similar to *AtAUX2-11*, and the previously named *FvAUX/IAA6*, and *Atl3F*, a potential response factor underlying a MAPKinase cascade. In addition, gene12691 is a protein phosphatase highly homologous to *Arabidopsis* ABA insensitive 2 (AtABI-2), which is negatively regulated by AtMYB20 (Cui et al., 2013).

### *FvMYB10* is a key activator in anthocyanin biosynthetic pathway

**Figure 5:**
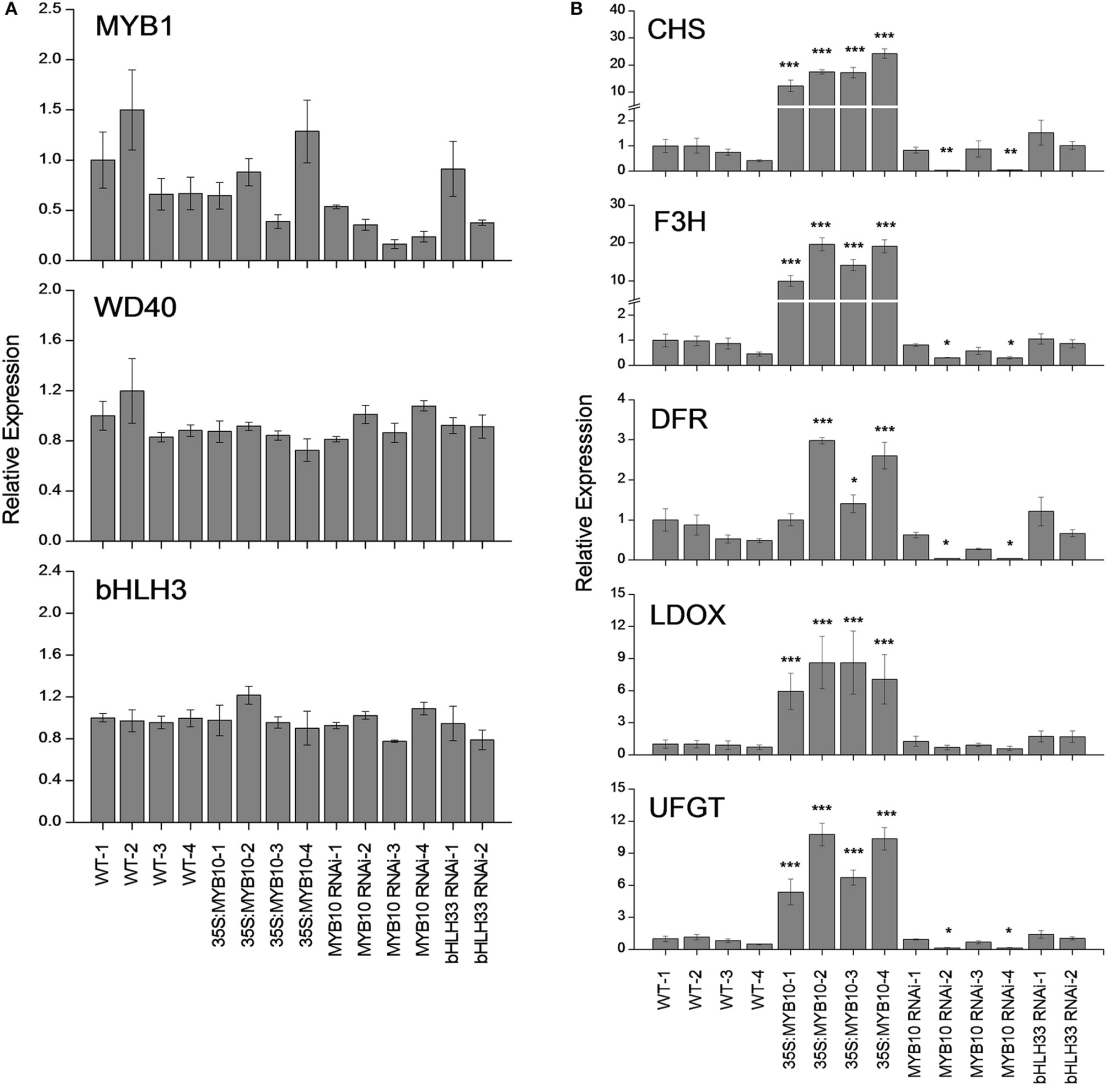
**Expression analysis of strawberry anthocyanin-related transcription factors and biosynthetic genes**. qPCR analysis of mature *F. vesca* fruit from four wild-type lines, four 35S:*FvMYB10* lines, four *FvMYB10* RNAi lines and two *FvbHLH33* RNAi lines. **(A)** *MYB1*, putative repressor; *bHLH3*, potential partner; *WD40*, *AtTTG1* like. **(B)** *CHS*, chalcone synthase; *F3H*, flavanone 3β-hydroxylase; *DFR*, dihydroflavonol 4-reductase; *LDOX*, leucoanthocyanidin dioxygenase; *UFGT*, uridine diphosphate (UDP)-glucose:flavonoid 3-*O*-glycosyltransferase. Transcript abundance was calculated relative to the geometric mean of *GAPDH*, *PP2a* and *UBC9*. Errors bars are the *SE* for three biological replicates. Statistical significance between transgenic lines and wild-type controls was determined by One-Way ANOVA: ^*^P < 0.05; ^**^P < 0.01; ^***^P < 0.001.

It has been previously reported that during fruit development *FaMYB10* and *FvMYB10* are strongly up-regulated in *F. ananassa* and *F. vesca* respectively (Lin-Wang et al., 2010). Similarly, the repressor *FaMYB1* is also up-regulated in *F. ananassa*, albeit to a lesser extent than the activator MYBs, while *FvMYB1* shows little change in *F. vesca*. In the present study, expression analysis was tested using qPCR of mature fruit from four independent 35S:*FvMYB10* over-expressing lines, four independent *FvMYB10* RNAi lines, two independent *FvbHLH33* RNAi lines and four wild-type lines (Figure 5). The expression of *FvMYB10* was greatly elevated from 6 to 27-fold in all *35S:FvMYB10* lines and reduced approximately five-fold in two *FvMYB10* RNAi lines (*FvMYB10* RNAi-2 and *FvMYB10* RNAi-4) compared with wild-type controls (Figure 2). The levels of transcripts encoding four anthocyanin biosynthetic genes Fv*CHS* (gene26826-v1.0-hybrid), *FvF3H* (gene14611-v1.0-hybrid), *FvDFR* (gene15174-v1.0-hybrid), *FvLDOX* (gene32347-v1.0-hybrid), and *FvUFGT* (gene12591-v1.0-hybrid) were examined among these lines, and showed elevations in all *35S:FvMYB10* lines and reductions in two *FvMYB10* RNAi lines (*FvMYB10* RNAi-2 and *FvMYB10* RNAi-4) compared with wild-type controls (Figure 5B). The expression of *FvbHLH33* decreased in two *FvbHLH33* RNAi lines (Figure 2). However, the reduction of *FvbHLH33* transcript did not have a significant impact on the levels of the transcripts of four other transcription factors and the five anthocyanin biosynthetic genes tested. The expression of *FvMYB10* was not knocked down in the other two independent *FvMYB10* RNAi lines (*FvMYB10* RNAi-1 and *FvMYB10* RNAi-3) and consequently the expression of anthocyanin biosynthetic genes was not affected (Figures 2, 5).

### Transient assay of strawberry MYB and bHLH activities

**Figure 6:**
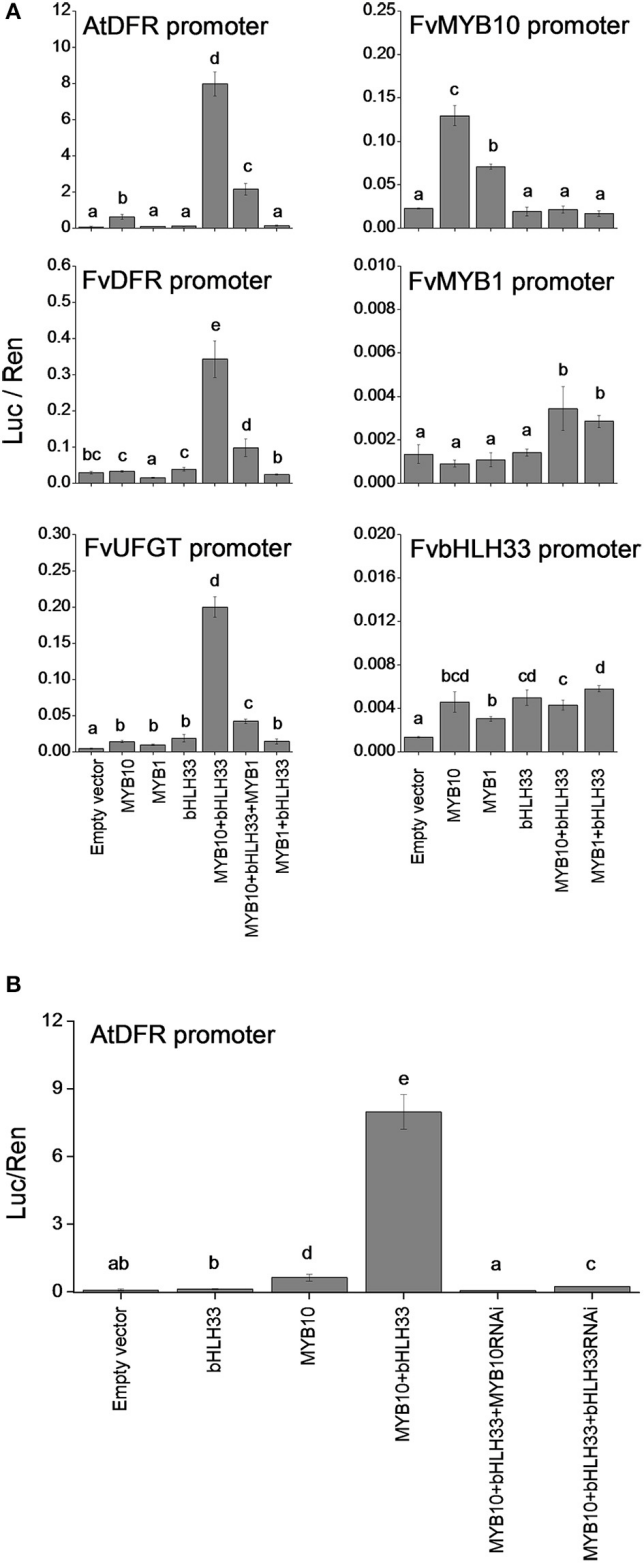
**Transient assay of the interaction between transcription factors and promoters of strawberry anthocyanin-related steps**. The dual luciferase assay, in *Nicotiana benthamiana* shows promoter activity expressed as a ratio of candidate promoter luciferase (Luc) to 35S Renilla (Ren). **(A)** FvMYB10 with FvbHLH33 activates the promoters of *AtDFR, FvDFR*, and *FvUFGT*, and FvMYB1 prevented the activation of these three promoters. The *FvMYB10* promoter was slightly induced by FvMYB10 and FvMYB1. No significant induction was observed in either the *FvMYB1* or *FvbHLH33* promoters when co-transformed with the transcription factors tested in the assay. **(B)** The activity of the *AtDFR* promoter was significantly reduced when either FvMYB10 RNAi or FvbHLH33 RNAi was co-transformed with FvMYB10 and FvbHLH33. Error bars are the *SE* for four replicate reactions. Statistical significance was determined by One-Way ANOVA; significant differences between means (LSD, *P* < 0.05) are indicated where letters (a, b, c, etc.) above the bar differ.

Dual luciferase assays were performed in *Nicotiana benthamiana* to examine FvMYB10 and FvMYB1 activity against the *Arabidopsis DFR* (At5g42800), *F. vesca DFR* and *UFGT* promoters and also to investigate interactions between FvMYBs and FvbHLHs. As shown in Figure 6A, FvMYB10 can activate the *Arabidopsis DFR*, *F. vesca DFR* (gene15174-v1.0-hybrid) and *UFGT* (gene12591-v1.0-hybrid) promoters, but only in the presence of FvbHLH33. This activation was strongly reduced when the repressor FvMYB1 was co-transformed with FvMYB10 and FvbHLH33.

In apple it has been shown that a 23-bp sequence motif in the *MdMYB10* promoter region is a target of the apple MdMYB10 protein itself and the number of repeats in this motif correlates with an increase in trans-activation by the MdMYB10 protein (Espley et al., 2009). This motif was not found in the promoter region of *FvMYB10*. However, the activity of the *FvMYB10* promoter was slightly induced by both FvMYB10 and FvMYB1 protein (Figure 6A). An analysis of cis-acting regulatory motifs within the *FvMYB10* promoter region showed a number of MYB-related elements (http://www.dna.affrc.go.jp/PLACE/) which could account for this, as well as bHLH-related E-boxes (Supplementary Table 7). There was a less significant induction observed in either the *FvMYB1* or *FvbHLH33* promoters when co-transformed with the transcription factors tested in the assay.

The activity of the *AtDFR* promoter was reduced when either FvMYB10 RNAi or FvbHLH33 RNAi was co-transformed with FvMYB10 and FvbHLH33 (Figure 6B). The *AtDFR* promoter was used in the transient RNAi assay because its activity was much stronger than the *F.vesca DFR* and *UFGT* promoters when induced by FvMYB10 and FvbHLH33 proteins.

### Expression of *FvMYB10* affects other members of the R2R3 MYB family

**Figure 7:**
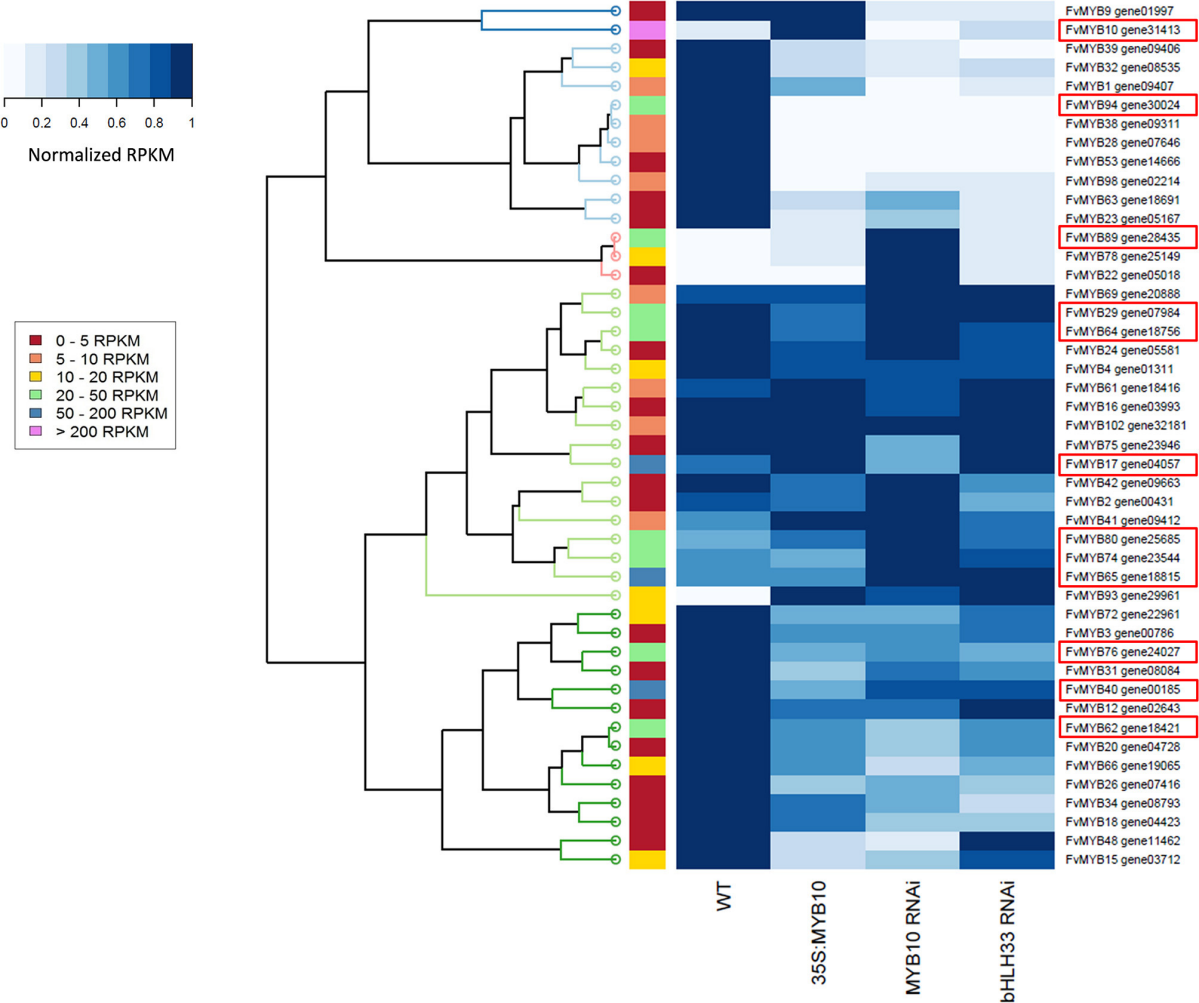
**Hierarchical clustering of MYB transcription factors differentially expressed in mature strawberry fruit from 35S:*FvMYB10*, *FvMYB10* RNAi, *FvbHLH33* RNAi, and wild-type control**. Heat map of 46 differentially expressed MYB transcription factors generated based on normalized RPKM values. Relative expression levels are represented as colors ranging from white (0: little or no expression) to dark blue (1: highest expression within four libraries) for each *MYB* gene. Four columns correspond to four RNA-seq libraries (mature fruit from 35S:*FvMYB10*, *FvMYB10* RNAi, *FvbHLH33* RNAi, and wild-type control). Each row represents each *MYB* gene. The color bar on the left indicates the range of the highest RPKM value within four libraries for each MYB gene. Clustering at the level of individual genes is presented by the dendrogram at the left of the heat map. The red box around the gene names represents a candidate gene showing that the RPKM value of the gene in at least one of the libraries greater than 20 and differential expression.

The MYB family of transcription factors has been reported to interact with each other in various networks. To ascertain if MYB10 co-regulates other MYBs, analysis of RNA-seq counts in the WT verses transgenic lines was made. Clustal analysis of MYBs with altered RPKM values are shown in Figure 7. A total of 12 MYBs, which showed values greater than 20 RPKM in at least one of the libraries, were affected by the over-expression of *MYB10*, or its knockdown, or knockdown of *bHLH33* (shown in red boxes, Figure 7).

Of interest was *FvMYB89*, an R2R3 MYB which was up-regulated by the knock-down of *MYB10*. Expression of *FvMYB89* was not detected in the WT at harvest, but increased to RPKM values of 20–50 when MYB10 was knocked down. FvMYB89 is homologous to AtMYB21, a gene normally expressed only in floral development, promoting petal and stamen growth (Reeves et al., 2012). The homolog of MYB89 is also affected in *F. ananassa* fruit when MYB10 is transiently knocked-down, where it was named *FaEOBII* because of its sequence similarity with *EOBII* (Emission of Benzenoids II) of *Petunia* (Medina-Puche et al., 2014). This TF has been previously characterized as a regulator of volatile phenylpropanoids in petunia flowers, such as phenylethyl alcohol, benzylbenzoate, eugenol, and isoeugenol (Spitzer-Rimon et al., 2010). FvMYB1, the repressor of anthocyanin biosynthesis showed a pattern similar to qPCR analysis (Figure 5), down slightly in the MYB10 RNAi lines. FvMYB17 is another candidate repressor, and is well expressed, and is up-regulated in 35S MYB10 lines, and down-regulated in knock out lines of MYB10.

### Analysis of fruit volatiles

**Figure 8:**
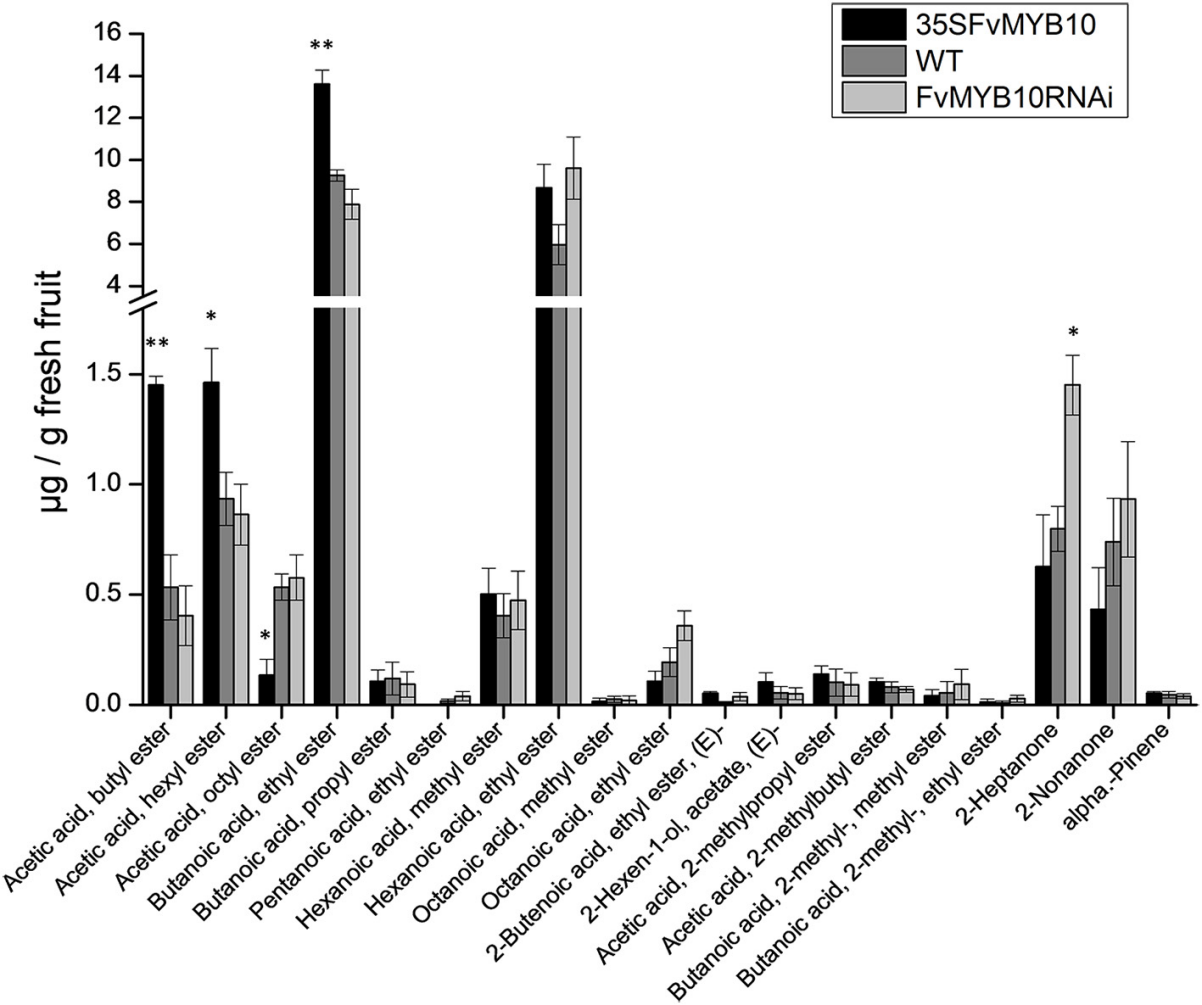
**Fruit volatiles of mature strawberry fruit from 35S:*FvMYB10*, *FvMYB10* RNAi, and wild-type plants**. GC-MS quantification of fruit volatiles. The acetic acids, butyl ester and hexyl ester and butanoic acid, ethyl ester were significantly higher, and acetic acid and octyl ester were significantly lower in 35S:*FvMYB10* than in the wild-type control and *FvMYB10* RNAi. *FvMYB10* RNAi fruit had the highest concentration of 2-heptanone. Statistical significance between transgenic lines and wild-type controls was determined by One-Way ANOVA: ^*^P < 0.05; ^**^P < 0.01.

Because of the observation that several floral-related MYBs were affected by the presence or absence of MYB10, an analysis of fruit volatiles was performed using GC-MS (7890A–5975C, Agilent Technologies). Esters have been reported as some of the most important sensory qualities for strawberry (Azodanlou et al., 2003). In our study, some significant differences were observed, particularly when comparing the over-expressing lines with the WT (Figure 8). The acetic acids, butyl ester and hexyl ester and butanoic acid, ethyl ester were all at higher concentrations, and acetic acid, and octyl ester were at lower concentrations in *35S:FvMYB10* lines. In contrast, the silenced lines showed little change from the WT, except for an increase in 2-heptanone. Whilst this compound has been identified in the white strawberry, *Fragaria chiloensis*, it is not necessarily a defining aroma volatile for white berries (Prat et al., 2014).

## Discussion

In the present study, a range of transgenic lines was used to test the specific roles of anthocyanin biosynthesis regulators in woodland strawberry. The use of independent over-expression and knock-down lines together with both targeted and global expression analysis provided strong evidence for these roles as well as suggesting the possibility of other seemingly unrelated effects such as volatile production and stress response.

The phenotypic evidence suggest no redundancy for MYB10; over-expressing lines had greatly increased anthocyanin concentrations whilst the two knock-down lines with the least *MYB10* expression had undetectable concentrations of both the major pigment contributing compounds, cyanidin glucoside, and pelargonidin glucoside. In contrast, knock-down of a potential partner of MYB10, bHLH33, had no phenotypic effect, suggesting a level of redundancy of the bHLH factors. It is clear that in a heterologous system, such as the promoter interaction assays presented here, bHLH33 is essential for regulation of anthocyanin biosynthesis steps. This does not appear to be the case in stably transformed strawberry where the lack of *bHLH33* expression does not affect anthocyanin concentrations.

The over-expression of *MYB10* did not alter other flavonoid concentrations in the fruit. Of all the metabolites tested, only p-coumaryl glucose was affected. This observation differs from that in apple, for example, where the over-expression of the homologous apple *MYB* resulted in increased flavon-3-ols and flavonols (Espley et al., 2013). This suggests a defined role for MYB10 in strawberry, limited to the anthocyanin branch of flavonoid production. Targeted gene expression analysis confirmed the function of MYB10 as the regulator of anthocyanin-related enzymes throughout the pathway from the first committed step, CHS, to the final glycosylation step, UFGT. In *F. ananassa* it has been reported that LDOX (also termed ANS) is not regulated by FaMYB10 (Medina-Puche et al., 2014). In *FaMYB10* silenced lines the transcript abundance was unchanged for *LDOX*, unlike for other genes in the anthocyanin pathway. The results here for *F. vesca* showed an increase in transcription of *LDOX* in *MYB10* over-expressing lines suggesting a role for MYB10 in *LDOX* transcription as with all the other anthocyanin biosynthesis genes tested. In *FvMYB10* silenced lines, *LDOX* shows a similar transcript level to the WT and is not statistically different, although the heavily silenced lines (lines 2 and 4) do have the least expression, concomitant with *CHS*, *F3H*, *DFR*, and *UFGT*. It appears then that FaMYB10 and FvMYB10 may have different roles in regulating *LDOX* transcription.

Data generated by RNA seq analysis confirmed MYB10 regulation of the anthocyanin pathway, and also revealed a more detailed picture of possible gene family members. These included possible duplications of *CHS*, *CHI*, *DFR*, and *UFGT* which all showed some level of transcription. Interestingly, the most differentially expressed gene was shown to be a glutathione S-transferase, homologous to a known anthocyanin transporter in *Arabidopsis*. In the list of genes both down-regulated in MYB10 over-expression lines, and up-regulated in white knock-down lines, there were several stress-related genes. This suggested that an absence of anthocyanins during fruit maturation leads to increase in cellular stress. Recently, increasing ascorbate in strawberries by transformation of the key regulatory gene GDP-galactose phosphorylase resulted in increased polyphenolics (Bulley et al., 2012). Ascorbate is a key compound in stress responses in plants supporting the idea that there may be a direct link between stress and polyphenolic concentrations.

Functional analysis of MYB10 using transient transformation of tobacco also showed that the TF was able to interact with and elevate the activity of key genes in the anthocyanin pathway, *DFR* and *UFGT*. However, this activation only occurred with the co-transformation of bHLH33. When the repressor TF MYB1 was also included with MYB10 and bHLH33 this activation was repressed. Transient transformation of bHLH33 on its own did not produce promoter activation. To test for auto-activation, *MYB10*, *MYB1*, and *bHLH33* promoters were all tested against their proteins. Autoregulation of anthocyanin-related MYBs has been previously demonstrated but this was due to a novel motif not present in the FvMYB10 promoter (Espley et al., 2009). The results shown here suggest that the *FvMYB10* promoter can be activated by MYB10 protein despite the apparent lack of auto-regulatory motifs found in the sequence. There are, however, a number of general MYB-related motifs within the isolated strawberry promoter region which may explain activation. It was interesting to note that transactivation was higher in the presence of MYB10 than of MYB1. The activation of *FvMYB10* with MYB1 suggests possible functionality of other anthocyanin-related MYBs. Interestingly, the MYB1 promoter, which also contains predicted MYB-related cis-acting elements, was apparently unresponsive to either the MYB10 or MYB1 proteins although there was some evidence for activation when co-infiltrated with bHLH33. Co-infiltration with bHLH33 produced no activation for the *FvMYB10* promoter, in contrast to similar experiments using apple MYB10 (Espley et al., 2007) where a combination of MYB and BHLH proteins had a stronger activation effect on the MYB10 promoter than MYB10 alone.

This work adds to the existing knowledge of anthocyanin regulation in strawberry and confirms that both cultivated and wild strawberry share similar anthocyanin regulation (Lin-Wang et al., 2010; Medina-Puche et al., 2014). With silencing of *MYB10*, there was some difference in the anthocyanin metabolites altered but this is likely to be due to the original anthocyanin composition in fruit from the two species. In both *FvMYB10* and *FaMYB10* transcription was shown to be fruit specific and correlated with anthocyanin accumulation throughout fruit ripening. Further, both *MYB10*s also appear to exert regulation on a broad range of genes involved in the anthocyanin pathway. However, silencing of *FaMYB10* had little or no effect on *LDOX* (*ANS*), which also appears to be the case for *FvMYB10*, where there was no statistical difference, in contrast to the other genes tested (*CHS*, *F3H*, *DFR*, and *UFGT)*. Conversely, stable over-expression of *FvMYB10* did lead to an increase in *LDOX* transcript levels suggesting that *FvMYB10* may have some regulating function, unless the increased transcript level is the result of increased pathway flux.

The transcriptome data showed that other MYBs, even those not associated with anthocyanin accumulation, were affected by over-expression of *MYB10*. Several of these MYBs are implicated in regulating volatile concentrations. In *MYB10* silenced *F.ananassa*, the expression of a volatile-related MYB, *FaEOBII* (*GENE28435*), was shown to be down-regulated (Medina-Puche et al., 2014). In *F. Vesca* the expression of this gene was higher in MYB10 knocked down fruit compared with WT or *FvMYB10* over-expressing lines (Figure 7). MYB TF regulation was also suggested for a *F. Ananassa* O-methyltransferase gene (*FaOMT*) involved in mesifurane production (Zorrilla-Fontanesi et al., 2012). The functional allele, as opposed to the non-functional allele, of *FaOMT* contains both MYB and bHLH-related binding domains in the promoter sequence which may account for the relatively high expression and consequent mesifurane content. Whilst this may not be relevant for *F. Vesca*, which has a relatively low level of *FvOMT* expression and low mesifurane concentration (Zorrilla-Fontanesi et al., 2012), it does further suggest the possibility for some role for MYBs in the regulation of strawberry volatile production. Further work is required to determine what, if any, direct effect the FvMYB10 protein may play on strawberry volatile production.

In summary, our study shows that both real-time quantitative PCR and transcriptomic analysis showed a strong correlation between the expression of *FvMYB10* and the concentrations of anthocyanin in 35S:*FvMYB10* lines, *FvMYB10* RNAi lines and wild-type controls. In contrast, *FvbHLH33*, which is a potential *bHLH* partner for *FvMYB10*, did not affect the anthocyanin pathway when knocked down using an RNAi construct. The results suggested the possible redundancy of *bHLH* partners in strawberry. In transient transactivation assays, *FvMYB10*, co-expressed with *FvbHLH33*, strongly activated the *AtDFR*, *FvDFR*, and *FvUFGT* promoters, while knocking down either *FvMYB10* or *FvbHLH33* significantly reduced the activity of the *AtDFR* promoter in *Nicotiana benthamiana* plants. FvMYB1, an R2R3 repressor MYB, proved to be a negative regulator of the strawberry anthocyanin biosynthetic pathway in transient assays.

There is a strong relationship with *MYB10* expression and other fruit ripening processes and it has also been demonstrated that *FaMYB10* expression and consequent anthocyanin concentration is effected by hormone levels in the fruit (Medina-Puche et al., 2014). It is likely that hormonal balance would similarly influence *FvMYB10*. The next challenge is to further elucidate the specific genes and hormonal cues that determine *MYB10* expression in strawberry.

### Conflict of interest statement

The authors declare that the research was conducted in the absence of any commercial or financial relationships that could be construed as a potential conflict of interest.

## References

[B1] Aharoni A. De Vos C. H. Wein M. Sun Z. Greco R. Kroon A.. (2001). The strawberry *FaMYB1* transcription factor suppresses anthocyanin and flavonol accumulation in transgenic tobacco. Plant J. 28, 319–332. 10.1046/j.1365-313X.2001.01154.x 11722774

[B2] Allan A. C. Hellens R. P. Laing W. A. (2008). MYB transcription factors that colour our fruit. Trends Plant Sci. 13, 99–102. 10.1016/j.tplants.2007.11.012 18280199

[B3] Azodanlou R. Darbellay C. Luisier J. L. Villettaz J. C. Amado R. (2003). Quality assessment of strawberries (*Fragaria* species). J. Agric. Food Chem. 51, 715–721. 10.1021/jf0200467 12537447

[B4] Baudry A. Caboche M. Lepiniec L. (2006). TT8 controls its own expression in a feedback regulation involving TTG1 and homologous MYB and bHLH factors, allowing a strong and cell-specific accumulation of flavonoids in *Arabidopsis thaliana*. Plant J. 46, 768–779. 10.1111/j.1365-313X.2006.02733.x 16709193

[B5] Blankenberg D. Gordon A. Von Kuster G. Coraor N. Taylor J. Nekrutenko A.. (2010). Manipulation of FASTQ data with galaxy. Bioinformatics 26, 1783–1785. 10.1093/bioinformatics/btq281 20562416 PMC2894519

[B6] Bulley S. Wright M. Rommens C. Yan H. Rassam M. Lin-Wang K.. (2012). Enhancing ascorbate in fruits and tubers through over-expression of the l-galactose pathway gene GDP-l-galactose phosphorylase. Plant Biotechnol. J. 10, 390–397. 10.1111/j.1467-7652.2011.00668.x 22129455

[B7] Butelli E. Titta L. Giorgio M. Mock H. P. Matros A. Peterek S.. (2008). Enrichment of tomato fruit with health-promoting anthocyanins by expression of select transcription factors. Nat. Biotechnol. 26, 1301–1308. 10.1038/nbt.1506 18953354

[B8] Chang S. Puryear J. Cairney J. (1993). A simple and efficient method for isolating RNA from pine trees. Plant Mol. Biol. Rep. 11, 113–116. 10.1007/BF0267046811725489

[B9] Cui M. H. Yoo K. S. Hyoung S. Nguyen H. T. Kim Y. Y. Kim H. J.. (2013). An *Arabidopsis* R2R3-MYB transcription factor, AtMYB20, negatively regulates type 2C serine/threonine protein phosphatases to enhance salt tolerance. FEBS Lett. 587, 1773–1778. 10.1016/j.febslet.2013.04.028 23660402

[B10] Davies K. M. Espley R. V. (2013). Opportunities and challenges for metabolic engineering of secondary metabolite pathways for improved human health characters in fruit and vegetable crops. N.Z. J. Crop Hortic. Sci. 41, 154–177 10.1080/01140671.2013.793730

[B11] Dubos C. Le Gourrierec J. Baudry A. Huep G. Lanet E. Debeaujon I.. (2008). MYBL2 is a new regulator of flavonoid biosynthesis in *Arabidopsis thaliana*. Plant J. 55, 940–953. 10.1111/j.1365-313X.2008.03564.x 18532978

[B12] Espley R. V. Bovy A. Bava C. Jaeger S. R. Tomes S. Norling C.. (2013). Analysis of genetically modified red-fleshed apples reveals effects on growth and consumer attributes. Plant Biotechnol. J. 11, 408–419. 10.1111/pbi.12017 23130849

[B13] Espley R. V. Brendolise C. Chagne D. Kutty-Amma S. Green S. Volz R.. (2009). Multiple repeats of a promoter segment causes transcription factor autoregulation in red apples. Plant Cell 21, 168–183. 10.1105/tpc.108.059329 19151225 PMC2648084

[B14] Espley R. V. Butts C. A. Laing W. A. Martell S. Smith H. McGhie T. K.. (2014). Dietary flavonoids from modified apple reduce inflammation markers and modulate gut microbiota in mice. J. Nutr. 144, 146–154. 10.3945/jn.113.182659 24353343

[B15] Espley R. V. Hellens R. P. Putterill J. Stevenson D. E. Kutty-Amma S. Allan A. C. (2007). Red colouration in apple fruit is due to the activity of the MYB transcription factor, MdMYB10. Plant J. 49, 414–427. 10.1111/j.1365-313X.2006.02964.x 17181777 PMC1865000

[B16] Fait A. Hanhineva K. Beleggia R. Dai N. Rogachev I. Nikiforova V. J.. (2008). Reconfiguration of the achene and receptacle metabolic networks during strawberry fruit development. Plant Physiol. 148, 730–750. 10.1104/pp.108.120691 18715960 PMC2556830

[B17] Fischer T. C. Mirbeth B. Rentsch J. Sutter C. Ring L. Flachowsky H.. (2014). Premature and ectopic anthocyanin formation by silencing of anthocyanidin reductase in strawberry (*Fragaria* × *ananassa*). New Phytol. 201, 440–451. 10.1111/nph.12528 24117941

[B18] Folta K. M. Davis T. M. (2006). Strawberry Genes and Genomics. Crit. Rev. Plant Sci. 25, 399–415 10.1080/07352680600824831

[B19] Folta K. M. Dhingra A. (2006). Invited review: transformation of strawberry: the basis for translational genomics in *Rosaceae*. In Vitro Cell. Dev. Biol. Plant 42, 482–490 10.1079/IVP2006807

[B20] Gleave A. P. (1992). A versatile binary vector system with a T-DNA organizational-structure conducive to efficient integration of cloned DNA into the plant genome. Plant Mol. Biol. 20, 1203–1207. 10.1007/BF00028910 1463857

[B21] Gonzalez A. Zhao M. Leavitt J. M. Lloyd A. M. (2008). Regulation of the anthocyanin biosynthetic pathway by the TTG1/bHLH/Myb transcriptional complex in *Arabidopsis* seedlings. Plant J. 53, 814–827. 10.1111/j.1365-313X.2007.03373.x 18036197

[B22] Griesser M. Hoffmann T. Bellido M. L. Rosati C. Fink B. Kurtzer R.. (2008). Redirection of flavonoid biosynthesis through the down-regulation of an anthocyanidin glucosyltransferase in ripening strawberry fruit. Plant Physiol. 146, 1528–1539. 10.1104/pp.107.114280 18258692 PMC2287331

[B23] Hannum S. M. (2004). Potential impact of strawberries on human health: a review of the science. Crit. Rev. Food Sci. Nutr. 44, 1–17. 10.1080/10408690490263756 15077879

[B24] Hellens R. P. Allan A. C. Friel E. N. Bolitho K. Grafton K. Templeton M. D.. (2005). Transient plant expression vectors for functional genomics, quantification of promoter activity and RNA silencing. Plant Methods 1:13. 10.1186/1746-4811-1-13 16359558 PMC1334188

[B25] Jaakola L. (2013). New insights into the regulation of anthocyanin biosynthesis in fruits. Trends Plant Sci. 18, 477–483. 10.1016/j.tplants.2013.06.003 23870661

[B26] Jin H. Cominelli E. Bailey P. Parr A. Mehrtens F. Jones J.. (2000). Transcriptional repression by AtMYB4 controls production of UV-protecting sunscreens in Arabidopsis. EMBO J. 19, 6150–6161. 10.1093/emboj/19.22.6150 11080161 PMC305818

[B27] Li H. Durbin R. (2009). Fast and accurate short read alignment with Burrows-Wheeler transform. Bioinformatics 25, 1754–1760. 10.1093/bioinformatics/btp324 19451168 PMC2705234

[B28] Lin-Wang K. Bolitho K. Grafton K. Kortstee A. Karunairetnam S. McGhie T. K.. (2010). An R2R3 MYB transcription factor associated with regulation of the anthocyanin biosynthetic pathway in *Rosaceae*. BMC Plant Biol. 10:50. 10.1186/1471-2229-10-50 20302676 PMC2923524

[B29] Lin-Wang K. Micheletti D. Palmer J. Volz R. Lozano L. Espley R.. (2011). High temperature reduces apple fruit colour via modulation of the anthocyanin regulatory complex. Plant Cell Environ. 34, 1176–1190. 10.1111/j.1365-3040.2011.02316.x 21410713

[B30] Medina-Puche L. Cumplido-Laso G. Amil-Ruiz F. Hoffmann T. Ring L. Rodriguez-Franco A.. (2014). MYB10 plays a major role in the regulation of flavonoid/phenylpropanoid metabolism during ripening of *Fragaria ananassa* fruits. J. Exp. Bot. 65, 401–417. 10.1093/jxb/ert377 24277278

[B31] Oosumi T. Gruszewski H. A. Blischak L. A. Baxter A. J. Wadl P. A. Shuman J. L.. (2006). High-efficiency transformation of the diploid strawberry (*Fragaria vesca*) for functional genomics. Planta 223, 1219–1230. 10.1007/s00425-005-0170-3 16320068

[B32] Pfaffl M. W. (2001). A new mathematical model for relative quantification in real-time RT-PCR. Nucleic Acids Res. 29, e45. 10.1093/nar/29.9.e45 11328886 PMC55695

[B33] Prat L. Espinoza M. I. Agosin E. Silva H. (2014). Identification of volatile compounds associated with the aroma of white strawberries (*Fragaria chiloensis*). J. Sci. Food Agric. 94, 752–759. 10.1002/jsfa.6412 24115051

[B34] Reeves P. H. Ellis C. M. Ploense S. E. Wu M. F. Yadav V. Tholl D.. (2012). A regulatory network for coordinated flower maturation. PLoS Genet. 8:e1002506. 10.1371/journal.pgen.1002506 22346763 PMC3276552

[B35] Rowan D. D. Cao M. Lin-Wang K. Cooney J. M. Jensen D. J. Austin P. T.. (2009). Environmental regulation of leaf colour in red 35S:PAP1 *Arabidopsis thaliana*. New Phytol. 182, 102–115. 10.1111/j.1469-8137.2008.02737.x 19192188

[B36] Salvatierra A. Pimentel P. Moya-Leon M. A. Herrera R. (2013). Increased accumulation of anthocyanins in *Fragaria chiloensis* fruits by transient suppression of FcMYB1 gene. Phytochemistry 90, 25–36. 10.1016/j.phytochem.2013.02.016 23522932

[B37] Schaart J. G. Dubos C. Romero De La Fuente I. Van Houwelingen A. M. De Vos R. C. Jonker H. H.. (2013). Identification and characterization of MYB-bHLH-WD40 regulatory complexes controlling proanthocyanidin biosynthesis in strawberry (*Fragaria* x *ananassa*) fruits. New Phytol. 197, 454–467. 10.1111/nph.12017 23157553

[B38] Shulaev V. Sargent D. J. Crowhurst R. N. Mockler T. C. Folkerts O. Delcher A. L.. (2011). The genome of woodland strawberry (*Fragaria vesca*). Nat. Genet. 43, 109–116. 10.1038/ng.740 21186353 PMC3326587

[B39] Snowden K. C. Simkin A. J. Janssen B. J. Templeton K. R. Loucas H. M. Simons J. L.. (2005). The Decreased apical dominance1/*Petunia hybrida* CAROTENOID CLEAVAGE DIOXYGENASE8 gene affects branch production and plays a role in leaf senescence, root growth, and flower development. Plant Cell 17, 746–759. 10.1105/tpc.104.027714 15705953 PMC1069696

[B40] Spitzer-Rimon B. Marhevka E. Barkai O. Marton I. Edelbaum O. Masci T.. (2010). EOBII, a gene encoding a flower-specific regulator of phenylpropanoid volatiles' biosynthesis in *Petunia*. Plant Cell 22, 1961–1976. 10.1105/tpc.109.067280 20543029 PMC2910970

[B41] Sun Y. Li H. Huang J. R. (2012). *Arabidopsis* TT19 functions as a carrier to transport anthocyanin from the cytosol to tonoplasts. Mol Plant 5, 387–400. 10.1093/mp/ssr110 22201047

[B42] Tamura K. Peterson D. Peterson N. Stecher G. Nei M. Kumar S. (2011). MEGA5: molecular evolutionary genetics analysis using maximum likelihood, evolutionary distance, and maximum parsimony methods. Mol. Biol. Evol. 28, 2731–2739. 10.1093/molbev/msr121 21546353 PMC3203626

[B43] Toufektsian M. C. De Lorgeril M. Nagy N. Salen P. Donati M. B. Giordano L.. (2008). Chronic dietary intake of plant-derived anthocyanins protects the rat heart against ischemia-reperfusion injury. J. Nutr. 138, 747–752. 18356330 10.1093/jn/138.4.747

[B44] Traka M. H. Mithen R. F. (2011). Plant science and human nutrition: challenges in assessing health-promoting properties of phytochemicals. Plant Cell 23, 2483–2497. 10.1105/tpc.111.087916 21803940 PMC3226206

[B45] Trapnell C. Williams B. A. Pertea G. Mortazavi A. Kwan G. Van Baren M. J.. (2010). Transcript assembly and quantification by RNA-Seq reveals unannotated transcripts and isoform switching during cell differentiation. Nat. Biotechnol. 28, 511–515. 10.1038/nbt.1621 20436464 PMC3146043

[B46] Wang M. Y. Macrae E. Wohlers M. Marsh K. (2011). Changes in volatile production and sensory quality of kiwifruit during fruit maturation in *Actinidia deliciosa* ‘Hayward’ and *A. chinensis* ‘Hort16A’. Postharvest Biol. Technol. 59, 16–24 10.1016/j.postharvbio.2010.08.010

[B47] Zorrilla-Fontanesi Y. Rambla J. L. Cabeza A. Medina J. J. Sanchez-Sevilla J. F. Valpuesta V.. (2012). Genetic analysis of strawberry fruit aroma and identification of *O-Methyltransferase FaOMT* as the locus controlling natural variation in mesifurane content. Plant Physiol. 159, 851–870. 10.1104/pp.111.188318 22474217 PMC3375946

