# Supplementary material for: Engineering the anthocyanin regulatory complex of strawberry (Fragaria vesca)
Source: Front Plant Sci. 2014 Nov 19;5:651. doi: 10.3389/fpls.2014.00651 (PMC4237049; doi:10.3389/fpls.2014.00651)
Supplement: Supplementary file 1 [file Table1.PDF]

Supplementary table 1. Oligonucleotide primer sequence for qPCR analysis.

Gene identifier	Primer sequence (5' to 3')
FvPP2a	TTTGAAGCGCCTTGCTGAAG<br>GGCAGATTGCACGCAGAAT
FvGAPDH	TCTTTGATGCCAAGGCTGGA<br>TCACACGGGAACTGTAACCC
FvUBC9	ATCTGCTCACTGTTGACGGA<br>AGCTCCTTGCTGTTGTCTCA
FvMYB10	TCAAATCAGGCTTAAACAGA<br>TTAAAGACCACCTGTTTCCT
FvMYB1	ATGAGGAAGCCCTGCTGCGA<br>AACGACGCAACCCTGCAGCC
FvbHLH3	ACCGAGTAGTAGCAGACTCCGTGGTAT<br>CCATCTGCCCATATTAACATCCCTTG
FvbHLH33	AATCCATGAGAGGGTGCCTGAGAAT<br>CAGCACCCCTTGTTGAGTTGTTGA
WD40	GACTTGAGGTACATGGCGACGATTT<br>TCATCCCCACCCGAGCAAATAT
CHS	TGACAAATCTATGATCAAGAAGCG<br>TCAAGTGAAGGTGCCATGTACTC
DFR	CACGATTCACGACATTGCGAAATT<br>GAACTCAAACCCCATCTCTTTCAGCTT
F3H	CCCTAAGGTGGCCTACAACCAAT<br>CTTCTTGCAAATCTCAGCGC
LDOX	GAAGTGCGTACCCAACTCCATCGT<br>ACCTTCTCCTTGTTGACGAGCCC
UFGT	CTAAGCAAAGGAAAGTTGAACGGAAT<br>TCCAACCGCAATGTGTTACAAA
