# Supplementary material for: Engineering the anthocyanin regulatory complex of strawberry (Fragaria vesca)
Source: Front Plant Sci. 2014 Nov 19;5:651. doi: 10.3389/fpls.2014.00651 (PMC4237049; doi:10.3389/fpls.2014.00651)
Supplement: Supplementary file 2 [file Table2.PDF]

Supplementary table 2. Abbreviations used for the polyphenols

**CyGlu**	cyanidin 3-O-glucoside	Anthocyanidins
**PgGlu**	pelargonidin 3-O-glucoside	
**PgMalGlu**	pelargonidin 3-O-(6”-malonyl-glucoside)	
**PgRut**	pelargonidin 3-O-rutinoside	
**Cy-rut**	cyanidin 3-O-rutinoside	
**ProCyB1**	procyanidin dimer B1	Proanthocyanidins
**ProCyB2**	Procyanidin dimer B2	
**ProCy3x**	Procyanidin trimer	
**Cat**	(+)-catechin	
**EC**	(-)-epicatechin	
**CGA**	chlorogenic acid	
**Cou-hex**	p-ccumaryl glucose	
**EA**	ellagic acid	
**IGluc**	isorhamnetin 3-O-glucoside	
**KGluc**	kaempferol -3-O-glucoside	
**Agm**	agrimoniin (ellagitannin)	
**Caf-hex**	caffeoyl glucoseCaffeic acid	
**QGluc**	quercetin 3-O-glucoside	
**QRut**	quercetin 3-O-rutinoside	
