# Supplementary figures and images for: Engineering the anthocyanin regulatory complex of strawberry (Fragaria vesca)
Source: Front Plant Sci. 2014 Nov 19;5:651. doi: 10.3389/fpls.2014.00651 (PMC4237049; doi:10.3389/fpls.2014.00651)

# Supplementary Figure 1. The LC-MS data for other polyphenols

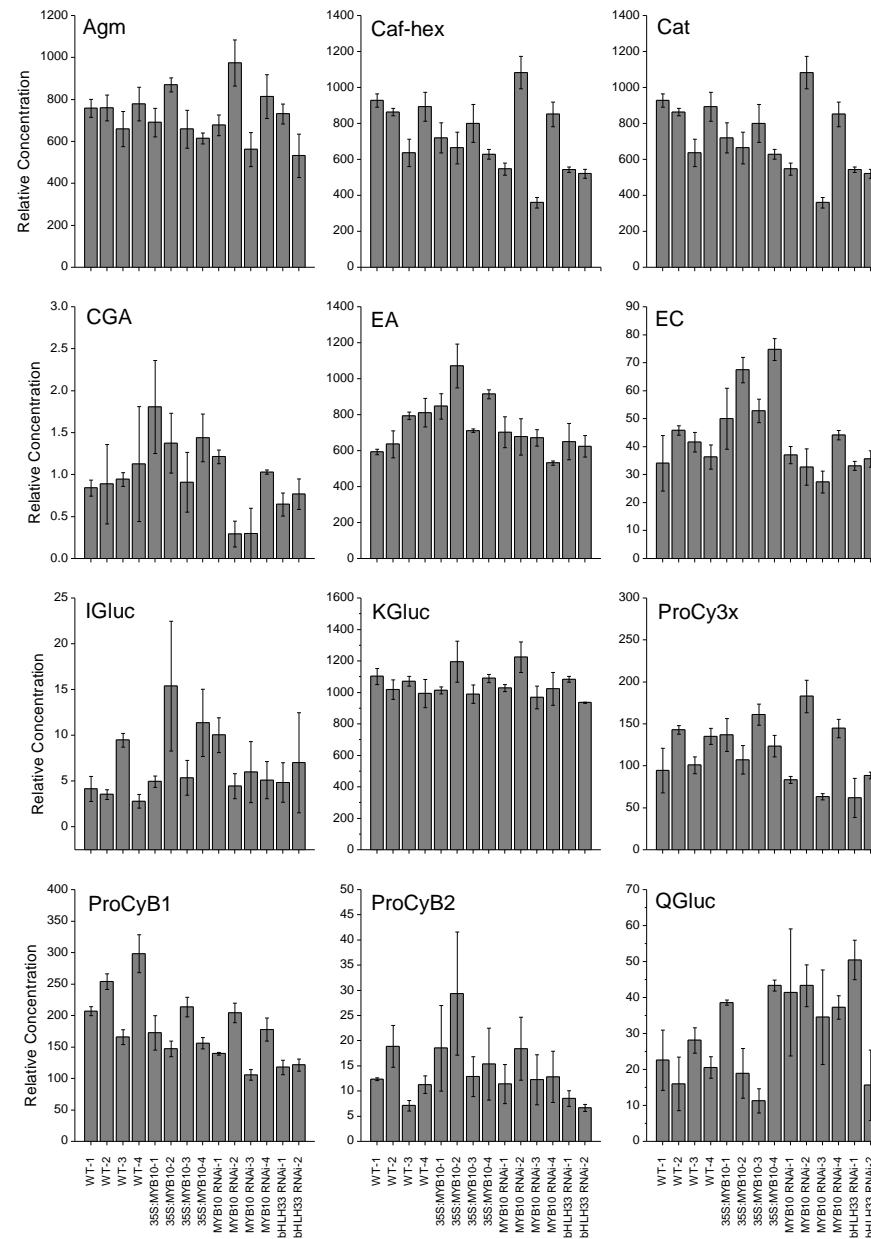

Supplement: Supplementary file 8 [file DataSheet1.PDF]
